# Interleukin-4 (IL-4) enhances and soluble interleukin-4 receptor (sIL-4R) inhibits histamine release from peripheral blood basophils and mast cells *in vitro* and *in vivo*

**DOI:** 10.1080/09629359791802

**Published:** 1997-04

**Authors:** B. Niggemann, T. Zuberbier, U. Herz, K. Enssle, U. Wahn, H. Renz

**Affiliations:** 1 Children's Hospital Virchow Clinic of Humboldt University Berlin Germany; 2 Institute for Clinical Chemistry and Biochemistry Virchow Clinic of Humboldt University Berlin Germany; 3 Department of Dermatology Virchow Clinic of Humboldt University Berlin Germany; 4 Behringwerke AG Marburg Germany

## Abstract

The aim of the study was to analyse the effect of interleukin-4 (IL-4) on allergen and anti-IgE mediated histamine release from basophils and human skin mast cells and to assess whether soluble recombinant interleukin-4 receptor (sIL4R) can inhibit these effects. Anti-IgE stimulated histamine release from peripheral blood basophils and mast cells of atopic donors was enhanced after preincubation with IL-4, whereas after preincubation with sIL-4R it was inhibited. These effects were even more pronounced when samples were stimulated with a clinically relevant allergen. In IL-4 preincubated skin mast cells, there was a similar enhancement of anti-IgE stimulated histamine release, which could again be inhibited by sIL-4R. The effects of IL-4 and sIL4R were dose- and time-dependent. Mice sensitized to ovalbumin and treated with soluble recombinant murine sIL-4R showed significantly reduced immediate-type cutaneous hypersensitivity responses compared with untreated mice. These *in vivo* effects were IgE independent, since there were no significant differences in total and allergen specific IgE/IgG1 antibody titres between treated and untreated mice. This indicates that IL4 exerts priming effects on histamine release by effector cells of the allergic response and that these effects are potently antagonized by soluble IL-4R both *in vitro* and *in vivo*.


Mediators of Inflammation, **6**, 111–118 (1997)

### Interleukin-4 (IL-4) enhances and soluble interleukin-4 receptor (sIL-4R) inhibits histamine release from peripheral blood basophils and mast cells *in vitro* and *in vivo*

B. Niggemann,[1,CA] T. Zuberbier,[3] U. Herz,[2] K. Enssle,[4] U. Wahn[1] and H. Renz[2]

[1]Children's Hospital, [2]Institute for Clinical Chemistry and Biochemistry, [3]Department of Dermatology, Virchow Clinic of Humboldt University, Berlin, Germany; [4]Behringwerke AG, Marburg, Germany

[CA]Corresponding Author
Tel: (+49) 30 450 66643
Fax: (+49) 30 450 66931

**THE aim of the study was to analyse the effect of interleukin-4 (IL-4) on allergen and anti-IgE mediated histamine release from basophils and human skin mast cells and to assess whether soluble recombinant interleukin-4 receptor (sIL-4R) can inhibit these effects. Anti-IgE stimulated histamine release from peripheral blood basophils and mast cells of atopic donors was enhanced after preincubation with IL-4, whereas after preincubation with sIL-4R it was inhibited. These effects were even more pronounced when samples were stimulated with a clinically relevant allergen. In IL-4 preincubated skin mast cells, there was a similar enhancement of anti-IgE stimulated histamine release, which could again be inhibited by sIL-4R. The effects of IL-4 and sIL-4R were dose- and time-dependent. Mice sensitized to ovalbumin and treated with soluble recombinant murine sIL-4R showed significantly reduced immediate-type cutaneous hypersensitivity responses compared with untreated mice. These *in vivo* effects were IgE independent, since there were no significant differences in total and allergen specific IgE/IgG1 antibody titres between treated and untreated mice. This indicates that IL-4 exerts priming effects on histamine release by effector cells of the allergic response and that these effects are potently antagonized by soluble IL-4R both *in vitro* and *in vivo*.**

**Key words:** Allergy, Basophil, Histamine release, IL-4, sIL-4R, Mast cell, Mice

### Introduction

Interleukin-4 (IL-4) plays a central role in allergic inflammation.[1] It acts as an important immunoglobulin isotype switch factor for IgE synthesis and is required for initiation and promotion of a TH2 T-cell response.[2–5] IL-4 stimulates expression of endothelial cell adhesion molecules on lymphocytes,[6] eosinophils, basophils and mast cells.[7–9] IL-4 production is increased in atopic dermatitis patients[10] and IgE synthesis induced by IL-4 is enhanced during seasonal allergen exposure.[11]

While there is clear evidence of IL-4 production by T-lymphocytes, some reports raise the possibility of IL-4 production by human mast cells[12,13] and basophils.[14] Furthermore, human mast cells express IL-4 receptors.[15] Mast cells and basophils represent important effector cells of the immediate-type allergic response which is characterized by the release of histamine and other mediators upon cross-linking of receptor bound IgE molecules by allergens. The expression of IL-4 receptors on these cells suggests that mast cells and basophils are sensitive to this cytokine. Since little is known about the interaction of IL-4 with effector cells, we examined the potential role of IL-4 on allergen and anti-IgE triggered histamine release from human basophils and skin mast cells. We examined further whether immunomodulation with an IL-4 antagonist would inhibit the IL-4 mediated effects. It has recently been shown that a recombinant soluble IL-4 receptor can inhibit many of the IL-4 mediated cell functions both *in vitro* and *in vivo*.[16,17] To examine the biological relevance of these findings, allergen sensitized mice were treated with a murine recombinant soluble IL-4 receptor and the immediate skin response was assessed.

### Material and Methods

### Patients

For experiments on basophils, blood was drawn from 11 adult atopic patients (six women, five men), aged 20–44 years (mean age 33 years).

© 1997 Rapid Science Publishers 

Total IgE ranged from 45 to 6650 kU/l (median 429 kU/l). Patients were sensitive to birch pollen (*Betula verrucosa*) (Bet v) and/or *Dermatophagoides pteronyssinus* (Der p). Sensitization was proven by specific IgE to birch and/or house-dust mite in serum (Pharmacia CAP-system, Uppsala, Sweden). Blood was taken over all the year except during the seasons April/May for birch- and September/October for house dust mite-sensitive patients. All patients suffered from allergic rhinoconjunctivitis, one patient from atopic dermatitis as well. All patients were without any anti-allergic medication for at least 4 weeks before drawing blood for the experiments. No patient had any infection during 2 weeks before the study.

Mast cells were prepared from normal human breast skin of six healthy female donors, aged 21–45 years (mean age 29 years), with no history of atopy who were undergoing plastic surgery for cosmetic reasons. All patients gave their informed consent. The study was approved by the local ethics committee.

### Reagents and buffer solutions

The following reagents were purchased: pipes, collagenase type 1a, hyaluronidase type I-S, chicken ovalbumin (OVA) grade V, (Sigma, Deisenhofen, Germany), Dextrane 6% (Braun, Melsungen, Germany), glucose 40% (Fresenius, Bad Homburg, Germany), perchloric acid (Merck, Darmstadt, Germany), allergen extract from Der p and Bet v (ALK, Copenhagen, Denmark), IL-4 (Dianova, Hamburg, Germany), DNAse (Boehringer, Mannheim, Germany), IgE, HSA (Pfrimmer, Erlangen, Germany), MEM (Earle's salts, fetal calf serum (FCS) (Gibco, Berlin, Germany), anti-IgE (Behring, Marburg, Germany); Pipes A (with 0.025% HSA), Pipes EDTA (Pipes A with 4 mM EDTA), and Pipes ACM (Pipes A with 2 mM $CaCl_2$ and 0.5 mM $MgCl_2$) were used in all experiments at a pH of 7.40. In experiments with human skin mast cells, MEM culture medium was supplemented with 2% FCS, 1% penicillin and streptomycin, 1% glutamine, 2% $MgSO_4$ and 10 µg/ml DNAse.

### Human and murine recombinant soluble IL-4 receptor

Soluble IL-4 receptor was prepared as previously described.[18] Briefly, using cDNA for the extracellular region of human and murine IL-4R,[19,20] recombinant soluble monomeric forms of IL-4R (human, rhuIL-4R and murine, muIL-4R) were constructed. The murine and human forms of rIL-4R were expressed in BHK cells. The proteins were purified by affinity chromatography using specific antibodies. Bioactivity, glycosylation and pharmacokinetics in mice were controlled for each lot of the molecules to achieve an uniform reactivity *in vitro* and *in vivo*.

### Preparation of basophil cultures from peripheral blood

Basophils were enriched and cultivated as previously described.[21] Briefly, peripheral blood leukocytes were obtained by spontaneous sedimentation for 60 min at room temperature. The percentage of viable basophils within the leukocyte preparation ranged between 6% and 11%. Cells were washed twice with Pipes A/EDTA and centrigued for 10 min. $1 \times 10^7$ leukocytes were placed in 4 ml plastic tubes (Sarstedt, Nümbrecht, Germany) in Pipes EDTA buffer. Cells were preincubated with IL-4 (100 to 5000 IU/ml), sIL-4R (1 to 200 µg/ml) or buffer in a water bath at 37°C for 2.5 h. After decanting supernatants and washing cells in Pipes A, Pipes ACM was added and cells were challenged with anti-IgE (30 and 100 IU/ml), Der p (30 and 100 SQ/ml) or Bet v (22 and 220 ng/ml). After a 30 min incubation at 37°C, cells were centrifuged for 15 min and supernatants were stored at −20°C until determination of histamine. All measurements were performed in duplicate.

### Preparation of human skin mast cells cultures

Mast cells were isolated by enzymatic dispersion, with slight modification as previously described.[22] Briefly, skin was cut into small squares (1–2 $mm^2$), dispersed in two 1-h cycles with collagenase and hyaluronidase (15 mg and 7.5 mg/g tissue respectively, 2.5 ml medium/g tissue). Undissociated tissue was removed by filtration with nylon gauze (150 µm) and thereafter the cells were washed twice in Pipes A. Viability of mast cells was determined by trypan blue staining and mast cell purity was assessed by toluidine blue staining. Viability of mast cells was > 95%. This procedure yielded $6–8 \times 10^5$ mast cells/g wet tissue with 3–5% mast cells of total nucleated cells. After collection of the dissociated cells, these were washed twice with Pipes A and then passively sensitized with IgE (1 µg/$10^6$ cells) at room temperature overnight. The cells were then incubated in Pipes ACM with 10–1000 ng/ml IL-4 for 5–15 min at 37°C in a water bath, or overnight in medium in an incubator. Aliquots of $10^4$ mast cells in 0.4 ml pipes ACM were challenged in duplicate with anti-IgE (1000, 2000 and 4000 IU/ml, with 0.417 IU precipitating 1 ng IgE WHO standard).

### Measurement of histamine release

Spontaneous histamine release, which was determined by the addition of Pipes ACM instead of stimuli, ranged between 5% and 10% of total histamine content. Total cellular histamine content was assayed by lysis of cell aliquots with 2% perchloric acid. After a 20 min incubation at 37°C, cells were centrifuged and supernatants were stored at −20°C until analysed.

Histamine was determined with an automated fluorometric analyser (Technicon, New York, USA) using a procedure previously described.[23] Results were only considered where spontaneous histamine release was less than 10% of total histamine content. More than 80% of our experiments fulfilled the criteria. For each result, spontaneous histamine release (incubation in Pipes ACM buffer) was subtracted and calculated as a percentage of total histamine content (= 100%) for each culture condition. The measurements were performed in duplicate.

### Allergic sensitization of mice

BALB/c mice (aged 6–10 weeks, from Bomholtgard, Denmark) were sensitized to OVA by nebulization of 1% ovalbumin (OVA) diluted in PBS. Sensitization was performed by exposure for 20 min every seventh day. As previously shown[24] and confirmed in this study, this procedure stimulated production of allergen-specific IgE/IgG1. After 4 weeks, mice were treated five times every other day by 20 min nebulization of 1 mg sIL-4R diluted in 7 ml PBS. Skin testing was performed 24 h after the last treatment. The study was approved by the local Animal Ethics Committee.

### Assessment of immediate-type skin tests response in mice

Intradermal skin tests (ST) were performed as previously described.[25] Briefly, abdominal skin was shaved and 50 µl of test solution was injected intradermally. PBS was used as negative control and compound 48/80 (Sigma, München, Germany) as positive control. Injection points were separated by at least 1.5 cm in order to avoid confluence of solutions. Allergen concentration was 50 µg/ml for ovalbumin. Resultant wheal formations were scored in a blinded fashion after 15–20 min by semiquantitative assessment of skin test reactivity. Reactions with a diameter ⩽ 1 mm were scored with 0 points, 2–4 mm with 1 point and reactions ⩾ 5 mm with 2 points. All animals developed positive wheal formations to compound 48/80, none reacted to PBS.

### Measurement of total and allergen-specific IgE/IgG1 antibody titres in serum samples

Analysis of total IgE and anti-OVA IgE/IgG1 antibody titres were determined by ELISA. Briefly, 96-well U-bottom, polystyrene microtitre plates (Greiner, Nürtingen, Germany) were coated either with anti-mouse IgE (10 µg/ml) (Pharmingen, San Diego, USA) or ovalbumin (20 µg/ml) Sigma, Deisenhofen, Germany) diluted in 0.05 M carbonate coating buffer, pH 9.6. Plates were incubated overnight, washed three times in washing buffer (PBS/0.05% plus Tween 20, pH 8.2) and blocked for 2 h at 37°C with blocking solution (2% BSA in PBS). After three washes with washing buffer, samples diluted in PBS/0.4% BSA were incubated for 2 h at 37°C. Washed plates were incubated with biotin-conjugated anti-mouse IgE/IgG1 monoclonal antibodies for 2 h at 37°C (2.5 µg/ml) (Pharmingen, San Diego, USA) followed by incubation with alkaline phosphate-conjugated streptavidin (1:500) (Jackson Immuno Research Laboratories, West Grove, USA) for 1 h at 37°C. Plates were developed with p-NPP substrate (0.5 mg/ml) (Sigma, Deisenhofen, Germany) and absorbance was measured at 405 nm.

### Statistical analysis

For statistical analysis, we used the Wilcoxon test for paired data, and Mann-Whitney *U*-test. For all numbers, standard error of mean is given. $P$ values < 0.05 were considered as statistically significant.

### Results

### Modulation of histamine release by human basophils

In order to investigate the effect of IL-4 on histamine release from human basophils, cells were stimulated with anti-IgE or the clinically relevant allergens. After preincubation with buffer alone, both anti-IgE and allergen enhanced histamine release in a dose-dependent manner, with a peak release at 100 IU/ml for anti-IgE (percentage of total histamine over background release 32.8% ± 4%). The optimal concentration of allergen ranged at 22 ng/ml for

Bet v (percentage of total histamine over background release 40.8%± 4%) and 100 standard quantity units (SQU)/ml for Der p (percentage of total histamine over background release 27.0%± 3%). Using concentrations lower than 22 ng/ml Bet v or 100 SQU/ml Der p, less pronounced histamine release was measured, which was not significantly different from controls (data not shown).

Cells were preincubated with IL-4 for 2.5 h followed by stimulation with optimal doses of anti-IgE and allergen. IL-4 enhanced anti-IgE triggered histamine release by 9.5%± 1.4% (Fig. 1). However, IL-4 by itself was unable to induce histamine release in the absence of any stimulus. A more pronounced effect was observed on allergen-triggered mediator release. Increases of histamine release by up to 35% were measured, with a percentage total histamine over background release of 19.0%± 6%. The reason why we converted the data into values of percentage stimulation/inhibition was the interindividual variability of the patients. We did not find major differences between Bet v and Der p effects in our culture system. For this reason, and because the individual groups would have been too small, we decided to present the results of both allergens together in one figure.

Since histamine release by peripheral blood basophils was analysed in the presence of cell subsets capable of secreting IL-4, and since

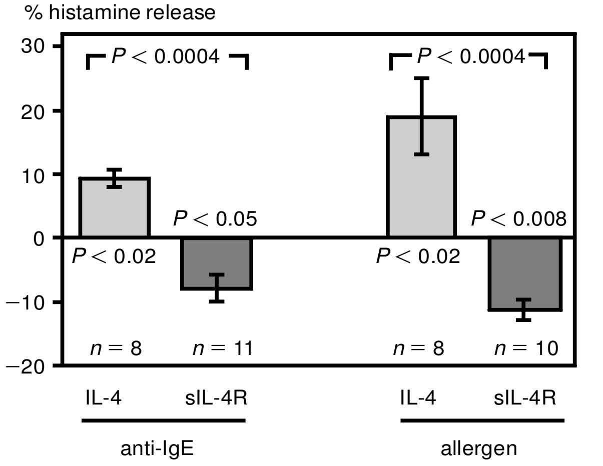

FIG. 1. Effect of IL-4 and sIL-4R on histamine release by peripheral blood basophils. Cells of atopic patients were preincubated with or without either IL-4 (5000 U/ml) or sIL-4R (200 µg/ml) for 2.5 h followed by stimulation with either anti-IgE or allergen. Mean percentage of total histamine over background release of anti-IgE stimulated cells was 32.8%, and 40.8% in allergen stimulated experiments. For each experiment, the percentage of stimulation/inhibition was calculated. Expressed are mean ± standard error of mean values. Experiments were performed with allergic patients; numbers are indicated in the figure. P-values around the baseline indicate statistical significance in relation to baseline values.

enhanced IL-4 production has been demonstrated by peripheral blood lymphocytes from atopic donors,[10,11,26] it was necessary to determine whether intrinsic ongoing IL-4 production contributed to the priming effect observed in the assay. A soluble recombinant human IL-4 receptor (sIL-4R) was added to cultures prior to stimulation with anti-IgE or allergen. Histamine release in peripheral blood basophils stimulated with anti-IgE was inhibited after preincubation with sIL-4R by −7.9 ± 6.5% (Fig. 1). An even more pronounced reduction was measured when cells were stimulated with allergen (Der p): sIL-4R inhibited histamine release by up to 20%, with a mean reduction of 11.3 ± 3.2% (Fig. 1). This data provides evidence that intrinsic ongoing IL-4 secretion by peripheral blood leukocytes exerts priming effects on histamine release by basophils.

Dose-response experiments showed that the effects of IL-4 (Fig. 2A) and sIL-4R (Fig. 2B) on both anti-IgE and allergen induced histamine release were dependent on the concentration. However, despite a clear tendency, results failed to reach statistical significance due to small numbers in some concentrations. Maximal augmentation of histamine release was found following preincubation with more than 1000 U/ml IL-4; sIL-4R at a concentration of > 100 µg/ml resulted in optimal inhibition of histamine release. To assess the specificity of these effects, cells were preincubated with a murine recombinant soluble IL-4R that does not bind to human IL-4. Incubation of leukocytes with murine sIL-4R in the same dose range was without any effects on anti-IgE or allergen-induced mediator release (data not shown).

Optimal effects were achieved when cells were preincubated with IL-4 and sIL-4R for about 2.5 h. Preincubation for a shorter period decreased the priming effect of IL-4 (data not shown). When cells were preincubated for a period longer than 3.5 h, spontaneous histamine release significantly ($P < 0.05$) increased and interfered with the assessment of IL-4/sIL-4R effects.

### Modulation of histamine release from human skin mast cells

To analyse whether IL-4 would also exert priming effects on other effector cells of the allergic reaction, we examined the effect of IL-4 on mast cells prepared from human non-atopic skin. Mast cells were presensitized with human IgE and *in vitro* histamine release was measured following stimulation with anti-IgE. There was a significant ($P < 0.05$) enhancement of

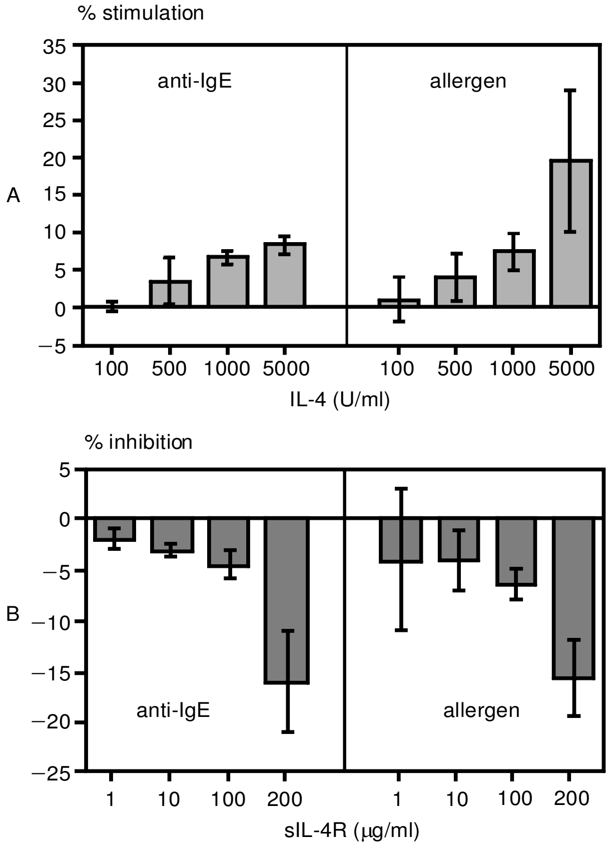

FIG. 2. Stimulation by IL-4 and inhibition by sIL-4R of histamine release. Cells from atopic patients were preincubated with or without IL-4 (100 to 5000 U/ml) (A) or sIL-4R (1–200 μg/ml) (B) for 2.5 h followed by stimulation with anti-IgE (100 IU/ml) and allergen (22 ng/ml Bet v and 100 SQ/ml Der p). A total of seven patients was analysed. For each donor, stimulation and inhibition of histamine release were compared with incubation with buffer alone and results are expressed in percentage changes. Expressed are mean ± standard error of mean values.

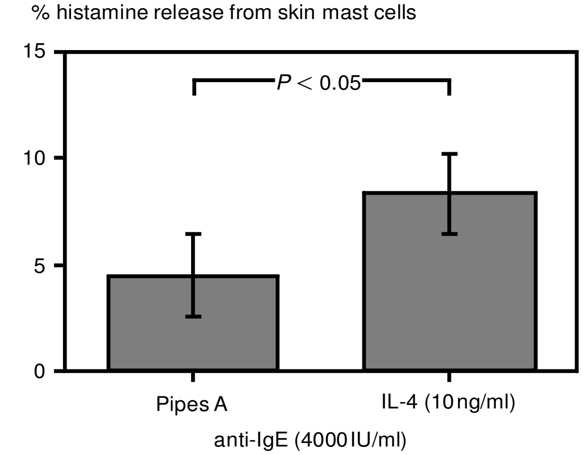

FIG. 3. IL-4 enhances anti-IgE induced histamine release from skin mast cells. Mast cells were prepared from the skin of non-atopic individuals (n = 6) and sensitized with human IgE. Cells were then preincubated with either Pipes A buffer or IL-4 (10 ng/ml) followed by stimulation with anti-IgE antibodies (4000 IU/ml). Expressed are mean ± standard error of mean values of histamine release. Histamine release was assessed and calculated as described in Material and Methods.

anti-IgE triggered histamine release (from 4.5% ± 1.9% to 8.3% ± 1.9%) following preincubation with IL-4 (Fig. 3). This could be blocked by simultaneous addition of sIL-4R ($n = 2$, data not shown).

### Inhibition of immediate-type cutaneous hypersensitivity responses by treatment with sIL-4R

The previous results indicate a priming effect of IL-4 and an inhibitory effect of sIL-4R on *in vitro* histamine release. The biological relevance of these findings was evaluated using an animal model of allergic sensitization and IgE production.[27] BALB/c mice were locally sensitized to ovalbumin via the airways. It has been shown that this procedure results in an increase of allergen-specific IgE/IgG1, development of positive skin test responses and enhanced airway responsiveness.[25] The mice were sensitized to OVA for 4 weeks followed by a brief period of treatment with murine sIL-4R also via the airways. The development of allergen specific cutaneous hypersensitivity responses was then assessed by intradermal injection of allergen as described above. As shown in Fig. 4, mice sensitized to OVA via airways and treated by the same route with sIL-4R developed significantly smaller wheal responses than mice that were

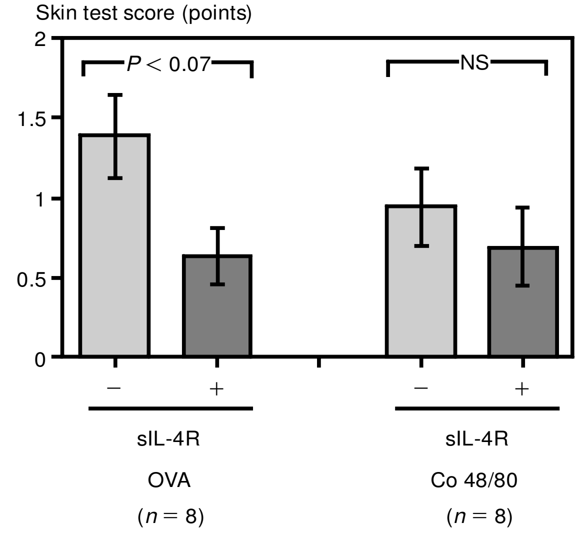

FIG. 4. Skin test response in sIL-4R treated and untreated mice. Mice were sensitized to OVA by aerosolization, then treated with sIL-4R via the same route (as described in Material and Methods) and skin tests were performed with OVA, compound 48/80 and PBS. Resultant wheal formations were scored in a blinded manner and results are expressed as mean ± standard error of mean values.

not treated with the drug ($P < 0.07$). Inhibition of *in vivo* mediator release was selective, since wheal formations stimulated by administration of compound 48/80 were not affected. Furthermore, these effects were not related to inhibition of IgE/IgG1 production by sIL-4R, since total and allergen specific IgE/IgG1 concentrations were the same in untreated OVA-sensitized animals (Table 1).

### Discussion

This study provides evidence that IL-4 primes effector cells of the allergic response to release histamine upon stimulation with allergen and anti-IgE. With allergen as the trigger factor for histamine release, mediator release was found to increase by up to 35%. These findings were supported by a similar and significant effect on the development of *in vivo* immediate-type cutaneous hypersensitivity responses. These results point to a novel function of IL-4.

IL-4 interferes with the allergic response at several levels of regulation: it is associated with the development of Th2 type T-lymphocytes and acts as a growth factor for Th2 cells;[2–5] it stimulates IgE synthesis[3–5] and is involved in mast cell activation.[7–9] IL-4 is produced by T-lymphocytes and effector cells including basophils and mast cells.[12,13] The results of this study indicate that the functional role of IL-4 in allergic inflammatory responses is not restricted to the development of pro-allergic T- and B-cell functions. It also interferes during the effector phase of the allergic response by priming mediator secretion from basophils and mast cells. We measured histamine release by these effector cells, since it represents an important and clinically relevant mediator of the early phase of the immediate-type allergic response. It is likely that secretion of other mediators which are released together with the prototypic mediator histamine is also enhanced under the influence of IL-4.

Since there is increasing evidence for the secretion of IL-4 by mast cells and basophils,[28,29] one can envisage a multidirectional activity of IL-4 during the development of immediate-type I allergic reactions. If IL-4 is released upon crosslinking of membrane-bound IgE antibodies, this source of IL-4 may prime neighbouring effector cells to release higher amounts of histamine (and other mediators). Therefore, IL-4 would potentiate the development of the allergic response in an autocrine and paracrine fashion. In our experiments it is possible that preactivated mast-cells and/or basophils were the source of IL-4 production.

Exactly how IL-4 primes effector cells requires further clarification. It is possible that it triggers the release of preformed mediators which are stored in cytoplasmic vesicles. Since IL-4 acts relatively quickly (the priming effects reached optimal levels within 2.5 h) it is unlikely that IL-4 increases histamine synthesis by regulation of transscriptional and/or translational events.

In skin mast cells, histamine releasability is generally lower than in basophils.[30] The enhancement of anti-IgE induced histamine release observed here was in the range of 4.5% mean histamine release before and 8.3% after pretreatment with IL-4. Although these differences may appear low, this represents an 80% increase of histamine release, which could be biologically relevant considering that the differences observed in anti-IgE induced basophil histamine release between normal controls and asthmatics appear low as well (median histamine release 20% vs. 26%).[31]

The effects of IL-4 were inhibited *in vitro* and *in vivo* by a recombinant soluble IL-4 receptor. We have shown in a previous study that treatment with sIL-4R can inhibit many IL-4 mediated functions under certain experimental conditions.[32] For example, when BALB/c mice were treated with sIL-4R simultaneously to allergen sensitization, it inhibited the development of IgE/IgG1 production and therefore the induction of immediate-type allergic reactions, including postive immediate-type skin test responses, and also increased airway responsiveness. We concluded from that study that the effects of sIL-4R treatment were related to a functional interference at the level of T–B cell interaction. To study whether sIL-4R would also act directly on the effector phase of the allergic response, we modified the sensitization and treatment protocol. Sensitized mice were treated with the receptor via nebulization for a short time prior to analysis of skin test reactivity. In the present study, the drug was delivered via the airways since it has recently been shown that local administration was superior to systemic treat-

**Table 1**. Serum total IgE, anti-OVA IgE and anti-OVA IgG1 in mice treated with sIL-4R in comparison with untreated animals (n = 16). Results (day 36) are expressed as mean ± SD

| | Treatment − sIL-4R | Treatment + sIL-4R | P |
|---|---|---|---|
| Total IgE (O.D.) | 0.31 ± 0.17 | 0.29 ± 0.19 | NS |
| Anti-OVA IgE (O.D.) | 0.27 ± 0.10 | 0.24 ± 0.10 | NS |
| Anti-OVA IgG1 (O.D.) | 0.41 ± 0.36 | 0.41 ± 0.35 | NS |

O.D. = optical density.

ment in terms of the inhibitory effects of immediate hypersensitivity responses.[33]

It is unlikely that the *in vivo* effects of sIL-4R in our study design were due to down-regulation of IgE/IgG1 production, because mice were already sensitized at the time the treatment was started and because the treatment was carried out over a short period of time. In addition, it is likely that Ig was already bound to IgE receptors on effector cells and it is suggested that receptor bound IgE has a long half-life time. In fact, measurement of IgE/IgG1 antibody titres indicated that there were no differences between sIL-4R treated and untreated mice. It is therefore likely that the effects observed in treated mice were related to a down-regulation of histamine release. SIL-4R treated mice still developed wheal formations immediately following allergen application into the skin, but the wheals were significantly smaller to a degree that reflects the results of the *in vitro* experiments. However, it should also be considered that IgE-mediated allergic sensitization to systemic allergen can occur in the absence of circulating IgE. In addition, a direct correlation exist between increased IgE production and mast cell activation.

In conclusion, this study provides evidence for a new functional activity of IL-4 as a cytokine which primes effector cells of the allergic response to release histamine upon stimulation with allergen. This activity underscores the important and multidirectional activities of this cytokine in the regulation of many aspects of the immunopathogenesis of allergic inflammatory responses. IL-4 not only controls the development of pro-allergic T-cell functions and triggers production of IgE, but also acts on effector cells of the allergic response. The biological relevance of these functions was demonstrated in sensitized mice which were treated with recombinant murine sIL-4R. The treatment study elucidated two important aspects: firstly, the development of *in vivo* immediate cutaneous hypersensitivity responses is directly controlled by IL-4, and, secondly, sIL-4R represents a potent immuno-pharmacological inhibitor for this (and other) functional activities of IL-4.

### References

1. Ricci M. IL-4: a key cytokine in atopy. *Clin Exp Allergy* 1994; **24**: 801–812.
2. Del Prete G, Maggi E, Parronchi P, Chrétien I, Tiri A, Macchia D, Ricci M, Banchereau J, De Vries J, Romagnani S. IL-4 is an essential factor for the synthesis induced in vitro by human T cell clones and their supernatants. *J Immunol* 1988; **140**: 4193–4198.
3. Jabara HH, Ahern DJ, Vercelli D, Geha RS. Hydrocortisone and IL-4 induce IgE isotype switching in human B cells. *J Immunol* 1991; **147**: 1557–1560.
4. Lebman DA, Coffman RL. Interleukin 4 causes isotype switching to IgE in T cell-stimulated clonal B cell cultures. *J Exp Med* 1988; **188**: 853–862.
5. Finkelman FD, Katona IM, Urban JF Jr, Holmes J, Ohara J, Tung AS, Sample JvG, Paul WE. IL-4 is required to generate and sustain in vivo IgE responses. *J Immunol* 1988; **141**: 2335–2341.
6. Galéa P, Thibault G, Lacord M, Bardos P, Lebranchu Y. IL-4, but not tumor necrosis factor-α, increases endothelial cell adhesiveness for lymphocytes by activating a cAMP-dependent pathway. *J Immunol* 1993: **151**: 588–596.
7. Schleimer RP, Sterbinsky SA, Kaiser J, Bickel CA, Klunk DA, Tomioka K, Newman W, Luskinskas FW, Gimbrone MA Jr, McIntire BW, Bochner BS. IL-4 induces adherence of human eosinophils and basophils but not neutrophils to endothelium. *J Immunol* 1992; **148**: 1086–1092.
8. Dubois GR, Bruijnzeel-Koomen CAFM, Bruijnzeel PLB. IL-4 induces chemotaxis of blood eosinophils from atopic dermatitis patients, but not from normal individuals. *J Invest Dermatol* 1994; **102**: 843–846.
9. Valent P, Bevec D, Maurer D, Besemer J, Di Padova F, Butterfield JH, Speiser W, Majdic O, Lechner K, Bettelheim P. Interleukin 4 promotes expression of mast cell ICAM-1 antigen. *Proc Natl Acad Sci* 1991; **88**: 3339–3342.
10. Jujo K, Renz H, Abe J, Gelfand EW, Leung DYM. Decreased interferon gamma and increased interleukin-4 production in atopic dermatitis promotes IgE synthesis. *J Allergy Clin Immunol* 1992; **90**: 323–331.
11. Gagnon R, Lian J, Boutin Y, Hébert J. Seasonal enhancement of IL-4 induced IgE synthesis by peripheral blood mononuclear cells of atopic patients. *Clin Exp Allergy* 1993; **23**: 498–503.
12. Bradding P, Feather IH, Howarth PH, Mueller R, Roberts JA, Britten K, Bews JPA, Hunt TC, Okayama Y, Heusser CH, Bullock GR, Church MK, Holgate ST. Interleukin 4 is localized to and released from human mast cells. *J Exp Med* 1992; **176**: 1381–1386.
13. Brunner T, Heusser CH, Dahinden CA. Human peripheral blood basophils primed by interleukin 3 (IL-3) produce IL-4 in response to immunoglobulin E receptor stimulation. *J Exp Med* 1993; **177**: 605–611.
14. Schroeder JT, MacGlashan DW, Kagey-Sobotka A, White JM, Lichtenstein LM. IgE-dependent IL-4 secretion by human basophils. *J Immunol* 1994; **153**: 1808–1817.
15. Valent P, Besemer J, Kishi K, Di Padova F, Geissler K, Lechner K, Bettelheim P. Human basophils express interleukin-4 receptors. *Blood* 1990; **76**: 1734–1738.
16. Maliszewski CR, Sato TA, van den Bos T, Waugh S, Dower SK, Slack J, Beckmann MP, Grabstein KH. Recombinant soluble receptors specifically inhibit IL-1 and IL-4 induced B cell activities in vitro. *J Immunol* 1990; **144**: 3028–3033.
17. Garrone P, Djossou O, Galizzi JP, Bancherau J. A recombinant extracellular domain of the human interleukin 4 receptor inhibits the biological effects of interleukin 4 on T and B lymphocytes. *Eur J Immunol* 1991; **21**: 1365–1369.
18. Renz H, Enssle K, Lauffer L, Kurrle R, Gelfand EW. Inhibition of allergen-induced IgE and IgG1 production by soluble IL-4 receptor. *Int Arch Allergy Immunol* 1995; **106**: 46–54.
19. Idzerda RL, March CJ, Mosley B, Lyman SD, Van den Bos T, Gimpel SD, Din WS, Grabstein KH, Widmer MD, Park LS, *et al.* Human interleukin-4 receptor confers biological responsiveness and defines a novel receptor superfamily. *J Exp Med* 1990; **171**: 861–873.
20. Mosley B, Beckmann MP, March CJ, Idzerda RL, Gimpel SD, Van den Bos T, Friend D, Alpert A, Anderson D, Jackson J, Wignall JM, Smith C, Gallis B, Sims JE, Urdal D, Widmer MB, Cosman D, Park LS. The murine interleukin-4 receptor: molecular cloning and characterization of secreted and membrane bound forms. *Cell* 1989; **59**: 335–348.
21. Wahn U, Ernsting M, Peterson J. Spontaneous histamine release from washed leucocytes and whole blood in atopic and non-atopic individuals. *Allergy* 1990; **45**: 109–114.
22. Lowman MA, Rees PH, Benyon RC, Church ML. Human mast cell heterogeneity: histamine release from mast cells dispersed from skin, lung, adenoids, tonsils, and colon in response to IgE-dependent and nonimmunologic stimuli. *J Allergy Clin Immunol* 1988; **81**: 590–597.
23. Siraganian RP. Refinements in the automated fluorometric histamine analysis system. *J Immunol Meth* 1975; **7**: 283–290.
24. Renz H, Smith HR, Henson JE, Ray BS, Irvin CG, Gelfand EW. Aerosolized antigen exposure without adjuvant causes increased IgE production and increased airway responsivesness in the mouse. *J Allergy Clin Immunol* 1992; **89**: 1127–1138.
25. Saloga J, Renz H, Lack G, Bradley KL, Greenstein JL, Larsen G, Gelfand EW. Development and transfer of immediate cutaneous hypersensitivity in mice exposed to aerosolized antigen. *J Clin Invest* 1993; **91**: 133–140.
26. Tang M, Kemp A, Varigos G. IL-4 and interferon-gamma production in children with atopic disease. *Clin Exp Immunol* 1993; **92**: 120–124.
27. Larsen GL, Renz H, Loader JE, Bradley KL, Gelfand EW. Airway response to electrical field stimulation in sensitized inbred mice. *J Clin Invest* 1992; **89**: 747–752.
28. Aoki I, Kinzer C, Shirai A, Paul WE, Klinman DM. IgE receptor positive non-B/non-T cells dominate the production of interleukin 4 and interleukin 6 in immunized mice. *Proc Natl Acad Sci USA* 1995; **92**: 2534–2538.

29. Schroeder JT, MacGlashan DW, Kagey-Sobotka A, White JM, Lichtenstein LM. IgE-dependent IL-4 secretion by human basophils. *J Immunol* 1994; **153**: 1808–1817.
30. Casolaro V, Spadaro G, Marone G. Human basophil releasability. VI. Changes in basophil releasability in patients with allergic rhinitis or bronchial asthma. *Am Rev Respir Dis* 1990; **142**: 1108–1111.
31. Lichey J, Zuberbier T, Luck W, Lau S, Wahn U. Influence of glycosphingolipids on the release of histamine and sulfidopeptide leukotrienes from human basophils. *Int Arch Allergy Immunol* 1994; **103**: 252–259.
32. Renz H, Bradley K, Enssle KH, Loader JE, Larsen GL, Gelfand EW. Prevention of the development of immediate hypersensitivity and airway responsiveness following in vivo treatment with soluble IL-4 receptor. *Int Arch Allergy Immunol* 1996; **109**: 167–176.
33. Lack G, Renz H, Saloga J, Bradley KL, Loader J, Leung DYM, Larsen G, Gelfand EW. Nebulized but not parenteral IFN-$\gamma$ decreases IgE production and normalizes airway function in a murine model of allergen sensitisation. *J Immunol* 1994; **152**: 2546–2554.

ACKNOWLEDGEMENTS. This work was supported by the 'Deutsche Forschungsgemeinschaft' (grant Re 737/4-1 and 4-2 to H.R.).

The authors thank Petra Dietl, Gabriele Schulz and Christiane Seib for excellent technical assistance.

**Received 5 November 1996;
accepted in revised form 28 November 1996**
